# Optical coherence tomography – A possible biomarker in early huntington’s disease

**DOI:** 10.1186/s42466-025-00421-z

**Published:** 2025-08-28

**Authors:** Clancy Cerejo, Elias Mandler, Federico Carbone, Gabriel Bsteh, Barbara Teuchner, Katarína Schwarzová, Marina Peball, Atbin Djamshidian, Klaus Seppi, Beatrice Heim

**Affiliations:** 1 Department of Neurology, Medical University of Innsbruck, Anichstrasse 35, Innsbruck, 6020 Austria; 2 Department of Neurology, Medical University of Vienna, Vienna, Austria; 3 Comprehensive Center for Clinical Neurosciences and Mental Health, Medical University of Vienna, Vienna, Austria; 4 Department of Ophthalmology and Optometry, Medical University of Innsbruck, Innsbruck, Austria; 5 Department of Neurology, Hospital Kufstein, Kufstein, Austria

**Keywords:** Huntington’s disease, Biomarkers, Optical coherence tomography, Olfactory dysfunction

## Abstract

**Objective:**

To assess the role of spectral domain Optical Coherence Tomography (OCT) as a biomarker in Huntington’s disease (HD).

**Methods:**

This cross-sectional study compared spectral domain OCT data, cognitive function, and olfactory function in HD patients and healthy controls (HC). HD patients were classified into Stage1 and Stage2 based on motor symptoms and functional capacity.

**Results:**

We recruited a total of 68 participants including 39HD patients (22 stage1, 17 stage2) and 29 age-matched HC. There were no significant differences in age and gender between the groups. Stage2 HD patients showed worse motor function (UHDRS-TMS 28.44 ± 18.13 vs. 13.74 ± 8.78, *p* = 0.002), functional capacity (UHDRS-TFC 8.13 ± 2.03 vs. 12.44 ± 0.99, *p* < 0.001), and lower scores on MMSE (27.36 ± 1.64 vs. 28.73 ± 1.74, *p* = 0.005 vs. 29.45 ± 0.91, *p* < 0.001) compared to stage1 HD patients and HC, respectively. Both stage1 and stage2 HD groups displayed significantly reduced macular retinal nerve fibre layer thickness (mRNFL) (33.45 ± 4.70, 31.90 ± 3.47 vs. 38.45 ± 5.00; *p* < 0.001) and ganglion cell-inner plexiform layer thickness (GCIPL) (71.63 ± 6.38, *p* = 0.007; 60.42 ± 4.67, *p* < 0.001 vs. 77.03 ± 8.40) as compared to HC. The retinal OCT parameters mRNFL and GCIPL correlated moderately with PIN_HD_ (*r*=-0.424, *r*=-0.513; *p* < 0.001), CAP (*r*=-0.425, *r*=-0.482; *p* < 0.001) and olfactory dysfunction for both smell identification (*r* = 0.446, *r* = 0.500; *p* < 0.001) and smell discrimination (*r* = 0.563, *r* = 0.467; *p* < 0.001).

**Conclusions:**

HD patients exhibit significantly thinner retinal ganglion cell inner plexiform layer and macular retinal nerve fibre layer compared to HC, even in the early phase of the disease. These findings suggest that OCT may serve as a valuable biomarker to monitor neurodegeneration at an early disease stage.

**Supplementary Information:**

The online version contains supplementary material available at 10.1186/s42466-025-00421-z.

## Introduction

Huntington’s disease (HD) is an autosomal dominant progressive neurodegenerative disorder caused by pathological CAG trinucleotides repeat expansion on chromosome 4 resulting in the production of mutant Huntingtin protein (mHTT) [1]. HD is predominantly characterised by motor, neuropsychiatric and cognitive symptoms [2]. At present, therapeutic strategies are limited to symptomatic management [3]. Numerous clinical trials are currently underway with the objective of developing disease-modifying therapies. The success of these trials depends on the availability of accurate and reliable biomarkers capable of tracking neurodegeneration over time. In the last decades, a substantial number of studies examining sensory functions in various neurodegenerative diseases including HD were published [4–15]. Optical Coherence Tomography (OCT) biomarkers are increasingly being investigated for the diagnosis, prognosis, and follow-up of neurodegenerative diseases [16–19]. Likewise, OCT may also be used to evaluate retinal parameters in HD patients. OCT is a non-invasive imaging technique used to detect retinal changes. It works similarly to ultrasound, but uses coherent light instead of sound waves, whose differential reflection and dispersion at layer boundaries is used to create high-resolution, 3-dimensional maps of the retina. This allows for precise measurement of individual retinal layer thicknesses [20]. OCT biomarkers such as thickness of the retinal nerve fibre layer (RNFL) and the combined macular ganglion cell and inner plexiform layer, ganglion cell-inner plexiform layer thickness (GCIPL) have been proven as a reliable surrogate marker of neuroaxonal damage in multiple sclerosis [21–24]. However, their value in other neurodegenerative diseases, especially the rare ones, is less well documented. In this study, we aimed to assess retinal changes in different disease stages of HD and to compare results with other clinical biomarkers.

## Methods

The study was approved by the local ethics committee of the Medical University of Innsbruck, Austria, and all participants provided written informed consent (AN1979 336/4.19 401/5.10 (4464a).

### Study participants

Participants were consecutively recruited during their routine follow up visit in a specialized outpatient clinic of the Medical University of Innsbruck, Department of Neurology, Austria. All HD patients had genetically confirmed HD. PIN_HD_ scores were calculated as described elsewhere [25]. All participants underwent clinical assessment by the same neurologist (B.H.), who is certified in the administration of the Unified Huntington’s Disease Rating Scale–Total Motor Score (UHDRS-TMS). Motor symptoms were evaluated using the Unified Huntington’s Disease Rating Scale (UHDRS)–total motor score (UHDRS-TMS). The Total Functional Capacity (TFC) scale, part of the Unified Huntington’s Disease Rating Scale, was used to assess disease stage. Accordingly, HD patients were categorized into stage1 and stage2 based on their functional capacities (UHDRS–total functional capacity, UHDRS-TFC) [26]. We did not include patients in more advanced disease stages as the increase in motor impersistence and chorea along with issues in optical fixation during the OCT measurements, could distort the results.

The following inclusion criteria had to be met to participate in the study: Positive genetic testing for HD, written consent of the subject, Mini Mental State Examination (MMSE) equal or over 24 points. Exclusion criteria comprised previous diagnoses of ophthalmological (i.e., myopia greater than − 6 dioptre’s, optic disc drusen, glaucoma, other abnormalities of the optic nerve head or the macula not attributable to HD), neurological, or drug-related causes of vision loss.

### Neuropsychological and olfactory testing

Participants underwent a cognitive test battery including the MMSE, the trail making test part A (TMT A) and B (TMT B), as well as the Symbol Digit Modalities Task (SDMT). Olfactory functioning was assessed using the 16-item Sniffin’ Sticks identification and discrimination test (Burghart Medizintechnik, Wedel, Germany).

### Optic coherence tomography

Optic coherence tomography (OCT) is an imaging technique used to detect retinal changes in ophthalmological and neurological conditions such as glaucoma or multiple sclerosis. Its operation is similar to that of an ultrasound device, but instead of sound waves, OCT emits light waves that are partially reflected at layer boundaries. Prior to this, light with a high bandwidth is split by a semi-transparent mirror, with one part directed onto the object and reflected there, while the other part is reflected at a mirror and serves as a reference value. The reflected light from both parts interferes with each other, and these interferences are measured by a spectrometer in Spectral Domain (SD)-OCT. With these interference data from many scans, a three-dimensional map of the retina can be generated, allowing the measurement of individual layer thicknesses. The advantages of OCT include its very high resolution of tissue in situ, achieved by the very short wavelength of light, and the fact that the examination is non-invasive and quick.

OCT imaging was performed by experienced neuro-ophthalmologists at the department of ophthalmology of the same institution using the same SD-OCT (Spectralis^®^, Heidelberg Engineering, No. 9468) without pupil dilatation in a dark room. The investigators performing the OCT were blinded to clinical parameters and vice versa.

A 20°×20° macular scan (512 A-scans, 25 B-scans, vertical alignment, ART: 16) centred on the macula was performed and the macular thickness map was analysed. All examinations were performed in accordance with the OSCAR-IB quality control criteria and described according to the APOSTEL 2.0 criteria [27, 28]. 

Semiautomated image processing was conducted using the built-in proprietary HEYEX^®^ software, Heidelberg Engineering, for automated layer segmentation with manual correction of obvious errors. Layer thicknesses of mRNFL and GCIPL were defined as the mean layer thickness of the four inner (3 mm ring) and outer quadrants (6 mm ring) of the circular grid centred around the foveola as defined by the Early Treatment Diabetic Retinopathy Study [29]. In addition, the sublayers within the GCIPL, ganglion cell layer (GCL) and the inner plexiform layer (IPL) were retrieved. By default, layer thicknesses were calculated as the mean of the values for both eyes.

## Statistics

Statistical analyses were performed using SPSS 29.0. To test for normal distribution, the Kolmogorov–Smirnov test was used. Parametric and non-parametric tests were used for statistical analysis depending on the distribution and the scale type of variables. The significance level was set at two-sided p-value of < 0.05 using a Bonferroni adjustment for group comparisons. Sum scores of the period of time to completion in the TMT part A and B were calculated per participant with a maximum of 240 seconds per task.

Exploratory analyses were conducted to assess the relationship between OCT measurements and clinical parameters, using Spearman correlation and multiple linear regression. The Bonferroni correction was applied post hoc to the results of multiple correlation analyses between OCT parameters and clinical variables, in order to adjust for multiple comparisons.

## Results

### Demographic characteristics (Table 1)

**Table 1:** Demographic characteristics

	Stage 1 HD	Stage 2 HD	HC	*P*-value
Number (n)	22	17	29	
Gender (m: f)	9:13	11:6	13:16	
Age (+/- SD)	47.65 ± 11.86	48.44 ± 17.59	42.10 ± 13.35	Stage 2 HD vs. Stage 1 HD *p* = 1.00 Stage 2 HD vs. HC *p* = 0.542 Stage 1 HD vs. HC *p* = 0.481
UHDRS-TMS (+/- SD)	13.74 ± 8.78	28.44 ± 18.13	n.a.	Stage 2 HD vs. Stage 1 HD *p* = 0.002**
UHDRS-TFC (+/- SD)	12.44 ± 0.99	8.13 ± 2.03	n.a.	Stage 2 HD vs. Stage 1 HD *p* < 0.001***
CAG repeats (+/- SD)	45.04 ± 3.80	45.81 ± 4.51	n.a.	Stage 2 HD vs. Stage 1 HD *p* = 0.568
PIN_HD_ (+/- SD)	2.24 ± 1.50	3.07 ± 1.51	n.a.	Stage 2 HD vs. Stage 1 HD *p* < 0.001
CAP score (+/- SD)	509.64 ± 87.80	515.43 ± 81.28	n.a.	Stage 2 HD vs. Stage 1 HD *p* < 0.001
MMSE (+/- SD)	28.73 ± 1.74	27.36 ± 1.64	29.45 ± 0.91	Stage 2 HD vs. Stage 1 HD *p* = 0.005** Stage 2 HD vs. HC *p* < 0.001*** Stage 1 HD vs. HC *p* = 0.117

Abbreviations: HD, patients with Huntington disease; HC, healthy controls; MMSE, Mini Mental State Examination; PIN_HD_, normed version of the Prognostic Index for Huntington’s Disease; SD, standard deviation; UHDRS, Unified Huntington’s Disease Rating Scale; UHDRS-TMS, Unified Huntington’s Disease Rating Scale – Total Motor Score; UHDRS-TFC, Unified Huntington’s Disease Rating Scale Total Functional Capacity; CAP score, CAG-Age-Product score.

The significance level is set at *p < 0.05. **p < 0.05, ***p ≤ 0.001. P-values of post-hoc comparisons are adjusted by Bonferroni correction for multiple comparisons. Disease staging: stage 1 (11 ≤ UHDRS-TFC ≤ 13); stage 2 (7 ≤ UHDRS-TFC ≤ 10); we did not include any patients from stages 3–5, as the increase in motor impersistence, along with issues in optical fixation during the OCT measurements, could distort the results.

We consecutively recruited 39 HD patients (78 eyes), of which 22 were in disease stage1 and 17 in disease stage2, along with 29 healthy controls (HC) (58 eyes).

The mean age in the HD (stage1, stage2) and control groups were 47.65 ± 11.86, 48.44 ± 17.59, and 42.10 ± 13.35 years respectively. There was no significant age-difference between the groups.

As expected, HD patients in stage2 showed significantly higher impairment of motor functions (UHDRS-TMS 28.44 ± 18.13, *p* = 0.002), functional capacity (UHDRS-TFC 8.13 ± 2.03, *p* < 0.001), and cognition (MMSE 27.36 ± 1.64, *p* = 0.005) compared to patients in stage1 (UHDRS-TMS 13.74 ± 8.78, UHDRS-TFC 12.44 ± 0.99, MMSE 28.73 ± 1.74).

### OCT metrics (Table 2)

**Table 2:** Results of olfactory testing, cognitive parameters and OCT measurements

	Stage 1 HD	Stage 2 HD	HC	*P*-value
Number (n)	22	17	29	
Odour identification (± SD) (corrected for MMSE and age)	10.87 ± 3.11	9.88 ± 2.90	15.00 ± 0.80	Stage 2 HD vs. Stage 1 HD *p* = 1.00 Stage 2 HD vs. HC *p* < 0.001*** Stage 1 HD vs. HC *p* < 0.001***
Odour discrimination (± SD) (corrected for MMSE and age)	9.62 ± 2.99	7.38 ± 3.89	15.07 ± 0.88	Stage 2 HD vs. Stage 1 HD *p* = 0.710 Stage 2 HD vs. HC *p* < 0.001*** Stage 1 HD vs. HC *p* < 0.001***
SDMT (± SD) (corrected for MMSE and age)	35.45 ± 15.94	24.71 ± 14.17	49.24 ± 9.81	Stage 2 HD vs. Stage 1 HD *p* = 1.00 Stage 2 HD vs. HC *p* = 0.015* Stage 1 HD vs. HC *p* = 0.024*
TMT-A (± SD) (corrected for MMSE)	45.35 ± 24.69	128.70 ± 82.25	28.07 ± 7.97	Stage 2 HD vs. Stage 1 HD *p* < 0.001*** Stage 2 HD vs. HC *p* < 0.001*** Stage 1 HD vs. HC *p* = 0.564
TMT-B (± SD) (corrected for MMSE and age)	113.65 ± 73.73	194.20 ± 72.04	53.93 ± 11.20	Stage 2 HD vs. Stage 1 HD *p* = 0.029* Stage 2 HD vs. HC *p* < 0.001*** Stage 1 HD vs. HC *p* = 0.009**
mRNFL (± SD) + (corrected for age)	33.45 ± 4.70	31.90 ± 3.47	38.45 ± 5.00	Stage 2 HD vs. Stage 1 HD *p* = 0.913 Stage 2 HD vs. HC *p* < 0.001*** Stage 1 HD vs. HC *p* < 0.001***
GCL (+/- SD) + (corrected for age)	39.76 ± 3.13	37.05 ± 3.24	42.21 ± 4.73	Stage 2 HD vs. Stage 1 HD *p* = 0.08 Stage 2 HD vs. HC *p* < 0.001*** Stage 1 HD vs. HC *p* = 0.020*
IPL (+/- SD) + (corrected for age)	32.92 ± 2.24	30.66 ± 2.00	35.74 ± 3.85	Stage 2 HD vs. Stage 1 HD *p* = 0.05 Stage 2 HD vs. HC *p* < 0.001*** Stage 1 HD vs. HC *p* = 0.039*
GCIPL (+/- SD) + (corrected for age)	71.63 ± 6.38	60.42 ± 4.67	77.03 ± 8.40	Stage 2 HD vs. Stage 1 HD *p* = 0.927 Stage 2 HD vs. HC *p* < 0.001*** Stage 1 HD vs. HC *p* = 0.007*

Abbreviations: HD, patients with Huntington disease; HC, healthy controls; PIN_HD_, normed version of the Prognostic Index for Huntington’s Disease; SD, standard deviation; SDMT, Symbol Digit Modalities Task; TMT A, Trail Making Test Part A; TMT B, Trail Making Test Part B; UHDRS, Unified Huntington’s Disease Rating Scale; UHDRS-TMS, Unified Huntington’s Disease Rating Scale – Total Motor Score; UHDRS-TFC, Unified Huntington’s Disease Rating Scale Total Functional Capacity. mRNFL, macular retinal nerve fibre layer; IPL, the inner plexiform layer; GCL, ganglion cell layer; GCIPL, ganglion cell-inner plexiform layer thickness. The significance level is set at *p < 0.05. **p < 0.05, ***p ≤ 0.001. P-values of post-hoc comparisons are adjusted by Bonferroni correction for multiple comparisons. Disease staging: stage 1 (11 ≤ UHDRS-TFC ≤ 13); stage 2 (7 ≤ UHDRS-TFC ≤ 10); we did not include any patients from stages 3–5, as the increase in motor impersistence, along with issues in optical fixation during the OCT measurements, could distort the results.

**Fig. 1:**
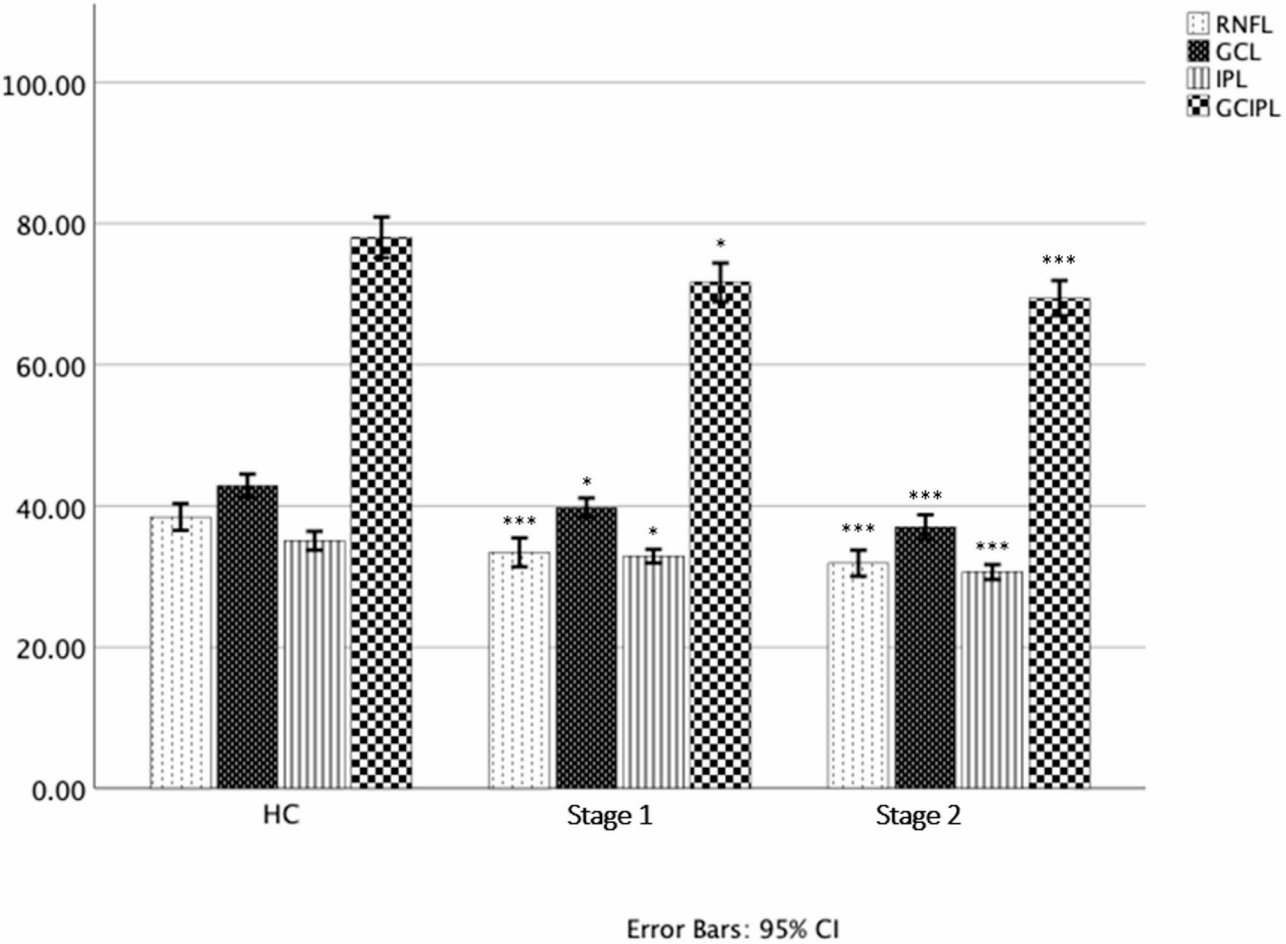
OCT measurements compared between groups

All included stage1 and stage2 HD patients successfully completed OCT measurements. No imaging artefacts were observed during the recordings, and no participants were excluded due to data quality issues or procedural intolerance. Both stage1 and stage2 HD groups had a significant decrease in total macular retinal nerve fibre layer (mRNFL) (33.45 ± 4.70, *p* < 0.001; 31.90 ± 3.47, *p* < 0.001 respectively) and GCIPL thickness (71.63 ± 6.38, *p* = 0.007; 60.42 ± 4.67, *p* < 0.001) as compared to HC (Fig. 1). Also, the GCL thickness (39.76 ± 3.13, *p* = 0.02; 37.05 ± 3.24 *p* < 0.001) and IPL thickness (32.92 ± 2.24, *p* = 0.039; 30.66 ± 2.00, *p* < 0.001 respectively) were significantly decreased in all HD patients further affirming the finding of GCIPL thickness.

The GCIPL had moderate correlation to the cognitive tests SDMT (*r* = 0.491, *p* < 0.001), TMT A (*r*=-0.404, *p* = 0.002), TMT B (*r*=-0.410, *p* = 0.001) and MMSE (*r* = 0.436, *p* < 0.001) in the HD group. The mRNFL had moderate correlation to SDMT (*r* = 0.399, *p* < 0.001) and TMT B (*r*=-0.391, *p* = 0.002) in the HD group.

Both mRNFL and GCIPL showed moderate correlation with the PIN_HD_ (*r*=-0.424 *r*=-0.513 respectively; *p* < 0.001) and CAG-Age-Product (CAP) score (*r*=-0.425, *r*=-0.482 respectively; *p* < 0.001); however, they showed no significant correlation with TMS (*r* = 0.053, *r*=-0.213 respectively) or TFC (*r*=-0.059, *r* = 0.132 respectively). Further, the OCT parameters mRNFL and GCIPL also had significant correlation with smell identification (*r* = 0.446, *r* = 0.500 respectively; *p* < 0.001) and smell discrimination (*r* = 0.563, *r* = 0.467 respectively; *p* < 0.001) (Additional file 1).

### Sniffin’ sticks test (Table 2)

In the Sniffin’ Stick battery, smell identification and discrimination were significantly reduced in both stage1 (10.87 ± 3.11, 9.62 ± 2.99 respectively) and stage2 HD (9.88 ± 2.90, 7.38 ± 3.89 respectively) patients as compared to the HC with all *p* < 0.001. These differences persisted even after correction for MMSE was applied.

When comparing the smell battery with the clinical parameters, both smell identification and discrimination had moderate to strong correlation with PIN_HD_ (*r*=-0.856, *r*=-0.756 respectively) and CAP (*r*=-0.826, *r*=-0.755 respectively) with *p* < 0.001. Both were also significantly correlated to cognitive parameters like MMSE (*r* = 0.567, *r* = 0.466 respectively), SDMT (*r* = 0.710, *r* = 0.594 respectively), TMT A (*r*=-0.631, *r*=-0.587 respectively) and TMT B (*r*=-0.638, *r*=-0.613 respectively) with all *p* < 0.001 (Additional file 1).

## Discussion

In the past few decades retinal OCT is emerging as a biomarker to track the progression of neurodegeneration and monitor treatment effectiveness especially for research purpose in various neurodegenerative diseases. The retina, as a direct extension of the central nervous system, is particularly of interest in evaluating neurodegenerative disorders as it can be easily, precisely, non-invasively and cost-effectively imaged by SD-OCT [20, 30, 31]. The RNFL containing unmyelinated axons of the optic nerve can serve as a mirror of axonal damage due to the neurodegenerative process within the central nervous system [30, 31]. The GCL, harbouring the neurons of the optic nerve, and the IPL, containing dendrites of GCL neurons, bipolar cell axons and amacrine cells, which are often analysed together as GCIPL for reasons of contrast within OCT imaging, reliably mirror neuronal damage within the optic system [21–24]. 

Retinal involvement in HD is not yet completely understood. Animal studies using R6 mouse models have indicated presence of progressive photoreceptor dysfunction, particularly cones, in the early disease stage with subsequent development of rod dysfunction, retinal remodelling and gliosis as the disease progresses [32–36]. Further, studies with transgenic mice have shown HD pathology selectively affecting retinal interneurons, causing inner retinal degeneration [32, 33, 35–37]. Studies in human HD patients have shown functional and structural changes in retina using VEP and OCT [7–15, 38–40]. In contrast to these finding, a post-mortem study in an advanced HD patient has shown no macroscopic or histological signs of neurodegeneration [41]. 

The aim of this single-centre study was to evaluate OCT biomarkers in different stages of manifest HD patients. All participants underwent a clinical test battery including cognitive tasks, smell identification and discrimination. We compared OCT parameters between stage1 and stage2 HD patients, and age matched controls.

The mRNFL and GCIPL were evaluated. In our study, mRNFL was significantly reduced in stage1 HD patients as compared to the HC, suggesting early structural changes in the retina even before severe neurodegeneration occurs. However, no significant difference was noted in between stage1 and stage2 HD groups. We think this could be because there was only a small difference between the stage1 and the stage2 HD groups; also, when comparing HC to all HD patients, the p-value of the mRNFL was 0.009. Among previous studies, Gulmez et al. has reported similar results with significant decrease in mRNFL thickness in HD as compared to the controls [11]. 

We found GCL, IPL and GCIPL thickness to be significantly decreased in all HD patients as compared to the HC. Similar findings are reported by Gulmez et al., while Di Maio et al. reported no difference in the ganglion cell complex parameters between the HD and HC [11, 13]. Several studies have examined OCT findings in pre-manifest HD patients. Among those, Svetozarskiy et al. reported a significant reduction in mean ganglion cell complex thickness in pre-manifest HD compared to healthy controls [12]. By contrast, Schmid et al. and Mazur-Michałek et al. found no significant differences in the average thickness of the ganglion cell–inner plexiform layer and ganglion cell complex, respectively, between pre-manifest HD and healthy control group [14, 42]. Notably, Schmid et al. suggested that the lack of significant differences in their study might be attributed to the fact that their pre-manifest HD cohort was, on average, more than 16 years from predicted disease onset.

In our cohort, OCT markers such as mRNFL and GCIPL thickness were significantly associated with disease severity as measured by the PIN_HD_ score and clinical disease stage, but not with TMS or TFC. This may reflect the limited sensitivity of TMS and TFC to detect subtle structural changes in early-stage patients, whereas PIN_HD_ and staging capture broader aspects of disease burden that align more closely with neurodegenerative retinal changes [43]. In addition, GCIPL had a significant correlation with the cognitive markers (SDMT, TMT A, TMT B, MMSE), suggesting that retinal thinning may be reflective of general cognitive abilities including attention, processing speed and executive function which are key areas affected in HD. Our findings are in line with a recent study, showing an association between retinal parameters and the cognitive markers [18]. 

The results from the Sniffin’ Sticks test indicate that both smell identification and discrimination abilities were significantly impaired in all HD patients compared to the HC group and these deficits persisted even after the adjustments for cognitive performance were done. Our results are consistent with other studies that have also reported olfactory dysfunction in HD patients [44, 45]. These findings suggest that olfactory impairment may be associated with underlying disease mechanisms in HD, and underscores the need for further research to explore its potential as a biomarker in HD.

A moderate to strong correlation between olfactory dysfunction and progression markers (PIN_HD_, CAP) was noted, indicating that olfactory dysfunction is closely related to the overall clinical burden in HD. Additionally, we observed a significant association between olfactory function and cognitive assessments (SDMT, TMT A, TMT B) suggesting that olfactory dysfunction is associated with cognitive deficits in HD and could serve as valuable indicators of cognitive decline in HD patients. Similar findings were reported in a study by Dintica et al. which evaluated the association between olfactory function and cognition in dementia free adults and reported that impaired olfactory function could predict a faster cognitive decline and also indicate neurodegeneration among these dementia-free older adults [46]. In addition, our findings are supported by a neuropathological study, which reported mHtt protein aggregates in the olfactory bulbs of HD patients [47]. 

Moreover, the significant association between OCT and olfactory dysfunction adds an intriguing dimension to the pathology of HD. Also, both the retinal thinning and olfactory dysfunction correlate significantly with cognitive decline and disease burden. These findings suggest a common mechanism involving neuronal degeneration in both the retina and the olfactory pathway.

Our study has certain limitations. The cross-sectional design of this study limits the ability to draw definitive conclusions about disease progression. Since HD is a rare disease, our sample size was small which prevents further generalization of the conclusions. Moreover, the utility of SD-OCT may be limited in the advanced HD stages as increased chorea or motor impersistence can lead to artefacts, segmentation errors, and reduce the reliability of retinal measurements. Using motion-correction SD-OCT systems and test-retest data could help address these issues. Also, studies with larger sample sizes possibly involving multicentre collaborations and longitudinal study designs could help to validate the utility of these biomarkers and to explore their role in predicting HD progression and therapeutic response.

## Conclusion

Our study contributes to the current evidence about the OCT biomarkers in HD patients. Our findings indicate that HD is associated with significant thinning of retinal layers. These reductions correlate moderately with cognitive performance, disease severity, and olfactory dysfunction, suggesting that OCT measurements could serve as useful indicators of neurodegeneration and cognitive decline even in early disease stages.

## Supplementary Information

Below is the link to the electronic supplementary material.

Supplementary Material 1: Correlation between different variables is available as ‘Additional file 1’.

## Data Availability

The datasets used and/or analysed during the current study are available from the corresponding author on reasonable request.

## Abbreviations

HD: Huntington’s disease
HC: Healthy controls
OCT: Optical Coherence Tomography
SD: Spectral Domain
mHTT: Mutant Huntingtin protein
RNFL: Retinal nerve fibre layer
GCL: Ganglion cell layer
IPL: Inner plexiform layer
GCIPL: Ganglion cell-inner plexiform layer thickness
UHDRS: Unified Huntington’s Disease Rating Scale
UHDRS-TMS: UHDRS total motor score
UHDRS-TFC: UHDRS total functional capacity
TMT A: Trail making test part A
TMT B: Trail making test part B
SDMT: Symbol Digit Modalities Task
MMSE: Mini Mental State Examination
CAP score: CAG-Age-Product score
