# Supplementary material for: Optical coherence tomography – A possible biomarker in early huntington’s disease
Source: Neurol Res Pract. 2025 Aug 28;7(1):61. doi: 10.1186/s42466-025-00421-z (PMC12395900; doi:10.1186/s42466-025-00421-z)
Supplement: Supplementary file 1 — Supplementary Material 1: Correlation between different variables is available as ‘Additional file 1’. [file 42466_2025_421_MOESM1_ESM.docx]

Supplementary Table 1: Correlation between different variables

|  | | | mRNFL | GCL | IPL | GCIPL | PIN_HD_ | CAP Score | SDMT | TMT-A | TMT-B | MMSE | Odour Identification | Odour Discrimination |
| --- | --- | --- | --- | --- | --- | --- | --- | --- | --- | --- | --- | --- | --- | --- |
|  | | |  |  |  |  |  |  |  |  |  |  |  |  |
| Spearman's rho | mRNFL | Correlation Coefficient | 1 | .645^**^ | .573^**^ | .549^**^ | -.424^**^ | -.425^**^ | .399^**^ | -.326^*^ | -.391^**^ | 0.203 | .446^**^ | .563^**^ |
|  |  | Sig. (2-tailed) | . | <.001 | <.001 | <.001 | <.001 | <.001 | <.001 | 0.012 | 0.002 | 0.104 | <.001 | <.001 |
|  |  | N | 68 | 68 | 68 | 68 | 68 | 68 | 68 | 59 | 59 | 65 | 68 | 53 |
|  | GCL | Correlation Coefficient | .645^**^ | 1 | .934^**^ | .882^**^ | -.503^**^ | -.462^**^ | .485^**^ | -.458^**^ | -.479^**^ | .461^**^ | .492^**^ | .569^**^ |
|  |  | Sig. (2-tailed) | <.001 | . | <.001 | <.001 | <.001 | <.001 | <.001 | <.001 | <.001 | <.001 | <.001 | <.001 |
|  |  | N | 68 | 68 | 68 | 68 | 68 | 68 | 68 | 59 | 59 | 65 | 68 | 53 |
|  | IPL | Correlation Coefficient | .573^**^ | .934^**^ | 1 | .880^**^ | -.493^**^ | -.441^**^ | .480^**^ | -.402^**^ | -.399^**^ | .415^**^ | .451^**^ | .474^**^ |
|  |  | Sig. (2-tailed) | <.001 | <.001 | . | <.001 | <.001 | <.001 | <.001 | 0.002 | 0.002 | <.001 | <.001 | <.001 |
|  |  | N | 68 | 68 | 68 | 68 | 68 | 68 | 68 | 59 | 59 | 65 | 68 | 53 |
|  | GCIPL | Correlation Coefficient | .549^**^ | .882^**^ | .880^**^ | 1 | -.513^**^ | -.482^**^ | .491^**^ | -.404^**^ | -.410^**^ | .436^**^ | .500^**^ | .467^**^ |
|  |  | Sig. (2-tailed) | <.001 | <.001 | <.001 | . | <.001 | <.001 | <.001 | 0.002 | 0.001 | <.001 | <.001 | <.001 |
|  |  | N | 68 | 68 | 68 | 68 | 68 | 68 | 68 | 59 | 59 | 65 | 68 | 53 |
|  | PIN_HD_ | Correlation Coefficient | -.424^**^ | -.503^**^ | -.493^**^ | -.513^**^ | 1 | .904^**^ | -.743^**^ | .730^**^ | .754^**^ | -.657^**^ | -.856^**^ | -.756^**^ |
|  |  | Sig. (2-tailed) | <.001 | <.001 | <.001 | <.001 | . | <.001 | <.001 | <.001 | <.001 | <.001 | <.001 | <.001 |
|  |  | N | 68 | 68 | 68 | 68 | 68 | 68 | 68 | 59 | 59 | 65 | 68 | 53 |
|  | CAP Score | Correlation Coefficient | -.425^**^ | -.462^**^ | -.441^**^ | -.482^**^ | .904^**^ | 1 | -.611^**^ | .635^**^ | .641^**^ | -.505^**^ | -.826^**^ | -.755^**^ |
|  |  | Sig. (2-tailed) | <.001 | <.001 | <.001 | <.001 | <.001 | . | <.001 | <.001 | <.001 | <.001 | <.001 | <.001 |
|  |  | N | 68 | 68 | 68 | 68 | 68 | 68 | 68 | 59 | 59 | 65 | 68 | 53 |
|  | SDMT | Correlation Coefficient | .399^**^ | .485^**^ | .480^**^ | .491^**^ | -.743^**^ | -.611^**^ | 1 | -.598^**^ | -.577^**^ | .620^**^ | .710^**^ | .594^**^ |
|  |  | Sig. (2-tailed) | <.001 | <.001 | <.001 | <.001 | <.001 | <.001 | . | <.001 | <.001 | <.001 | <.001 | <.001 |
|  |  | N | 68 | 68 | 68 | 68 | 68 | 68 | 68 | 59 | 59 | 65 | 68 | 53 |
|  | TMT-A | Correlation Coefficient | -.326^*^ | -.458^**^ | -.402^**^ | -.404^**^ | .730^**^ | .635^**^ | -.598^**^ | 1 | .754^**^ | -.360^**^ | -.631^**^ | -.587^**^ |
|  |  | Sig. (2-tailed) | 0.012 | <.001 | 0.002 | 0.002 | <.001 | <.001 | <.001 | . | <.001 | 0.005 | <.001 | <.001 |
|  |  | N | 59 | 59 | 59 | 59 | 59 | 59 | 59 | 59 | 59 | 59 | 59 | 53 |
|  | TMT-B | Correlation Coefficient | -.391^**^ | -.479^**^ | -.399^**^ | -.410^**^ | .754^**^ | .641^**^ | -.577^**^ | .754^**^ | 1 | -.569^**^ | -.638^**^ | -.613^**^ |
|  |  | Sig. (2-tailed) | 0.002 | <.001 | 0.002 | 0.001 | <.001 | <.001 | <.001 | <.001 | . | <.001 | <.001 | <.001 |
|  |  | N | 59 | 59 | 59 | 59 | 59 | 59 | 59 | 59 | 59 | 59 | 59 | 53 |
|  | MMSE | Correlation Coefficient | 0.203 | .461^**^ | .415^**^ | .436^**^ | -.657^**^ | -.505^**^ | .620^**^ | -.360^**^ | -.569^**^ | 1 | .567^**^ | .466^**^ |
|  |  | Sig. (2-tailed) | 0.104 | <.001 | <.001 | <.001 | <.001 | <.001 | <.001 | 0.005 | <.001 | . | <.001 | <.001 |
|  |  | N | 65 | 65 | 65 | 65 | 65 | 65 | 65 | 59 | 59 | 65 | 65 | 53 |
|  | Odour Identification | Correlation Coefficient | .446^**^ | .492^**^ | .451^**^ | .500^**^ | -.856^**^ | -.826^**^ | .710^**^ | -.631^**^ | -.638^**^ | .567^**^ | 1 | .778^**^ |
|  |  | Sig. (2-tailed) | <.001 | <.001 | <.001 | <.001 | <.001 | <.001 | <.001 | <.001 | <.001 | <.001 | . | <.001 |
|  |  | N | 68 | 68 | 68 | 68 | 68 | 68 | 68 | 59 | 59 | 65 | 68 | 53 |
|  | Odour Discrimination | Correlation Coefficient | .563^**^ | .569^**^ | .474^**^ | .467^**^ | -.756^**^ | -.755^**^ | .594^**^ | -.587^**^ | -.613^**^ | .466^**^ | .778^**^ | 1 |
|  |  | Sig. (2-tailed) | <.001 | <.001 | <.001 | <.001 | <.001 | <.001 | <.001 | <.001 | <.001 | <.001 | <.001 | . |
|  |  | N | 53 | 53 | 53 | 53 | 53 | 53 | 53 | 53 | 53 | 53 | 53 | 53 |

**. Correlation is significant at the 0.01 level (2-tailed).

*. Correlation is significant at the 0.05 level (2-tailed).

Abbreviations: mRNFL, macular retinal nerve fiber layer; IPL, inner plexiform layer; GCL, ganglion cell layer; GCIPL, ganglion cell-inner plexiform layer thickness; PIN_HD_, normed version of the Prognostic Index for Huntington's Disease; CAP score, CAG-Age-Product; SDMT, Symbol Digit Modality Task; TMT A, Trail Making Test Part A; TMT B, Trail Making Test Part B; MMSE, Mini Mental State Examination.

Supplementary Table 2: Correlation between different variables

|  |  |  | TFC | mRNFL | GCIPL | TMS | CAP Score | PIN_HD_ | Age |
| --- | --- | --- | --- | --- | --- | --- | --- | --- | --- |
| TFC | Correlation Coefficient |  | 1 | -0.059 | 0.132 | -.606^**^ | -0.005 | -.340^*^ | -0.121 |
|  | Sig. (2-tailed) |  | . | 0.723 | 0.424 | <.001 | 0.976 | 0.034 | 0.462 |
|  | N |  | 39 | 39 | 39 | 39 | 39 | 39 | 39 |
| mRNFL | Correlation Coefficient |  | -0.059 | 1 | .549^**^ | 0.053 | -.425^**^ | -.424^**^ | -0.013 |
|  | Sig. (2-tailed) |  | 0.723 | . | <.001 | 0.751 | <.001 | <.001 | 0.913 |
|  | N |  | 39 | 68 | 68 | 39 | 68 | 68 | 68 |
| GCIPL | Correlation Coefficient |  | 0.132 | .549^**^ | 1 | -0.213 | -.482^**^ | -.513^**^ | -0.141 |
|  | Sig. (2-tailed) |  | 0.424 | <.001 | . | 0.193 | <.001 | <.001 | 0.25 |
|  | N |  | 39 | 68 | 68 | 39 | 68 | 68 | 68 |
| TMS | Correlation Coefficient |  | -.606^**^ | 0.053 | -0.213 | 1 | .461^**^ | .861^**^ | 0.112 |
|  | Sig. (2-tailed) |  | <.001 | 0.751 | 0.193 | . | 0.003 | <.001 | 0.496 |
|  | N |  | 39 | 39 | 39 | 39 | 39 | 39 | 39 |
| CAP Score | Correlation Coefficient |  | -0.005 | -.425^**^ | -.482^**^ | .461^**^ | 1 | .904^**^ | 0.223 |
|  | Sig. (2-tailed) |  | 0.976 | <.001 | <.001 | 0.003 | . | <.001 | 0.067 |
|  | N |  | 39 | 68 | 68 | 39 | 68 | 68 | 68 |
| PIN_HD_ | Correlation Coefficient |  | -.340^*^ | -.424^**^ | -.513^**^ | .861^**^ | .904^**^ | 1 | 0.222 |
|  | Sig. (2-tailed) |  | 0.034 | <.001 | <.001 | <.001 | <.001 | . | 0.069 |
|  | N |  | 39 | 68 | 68 | 39 | 68 | 68 | 68 |
| Age | Correlation Coefficient |  | -0.121 | -0.013 | -0.141 | 0.112 | 0.223 | 0.222 | 1 |
|  | Sig. (2-tailed) |  | 0.462 | 0.913 | 0.25 | 0.496 | 0.067 | 0.069 | . |
|  | N |  | 39 | 68 | 68 | 39 | 68 | 68 | 68 |
| Odour Identification | Correlation Coefficient |  | .366^*^ | .446^**^ | .500^**^ | -.539^**^ | -.826^**^ | -.856^**^ | -0.232 |
|  | Sig. (2-tailed) |  | 0.022 | <.001 | <.001 | <.001 | <.001 | <.001 | 0.057 |
|  | N |  | 39 | 68 | 68 | 39 | 68 | 68 | 68 |
| Odour Discrimination | Correlation Coefficient |  | .406^*^ | .563^**^ | .467^**^ | -.528^**^ | -.755^**^ | -.756^**^ | -.362^**^ |
|  | Sig. (2-tailed) |  | 0.049 | <.001 | <.001 | 0.008 | <.001 | <.001 | 0.008 |
|  | N |  | 24 | 53 | 53 | 24 | 53 | 53 | 53 |
| SDMT | Correlation Coefficient |  | .410^**^ | .399^**^ | .491^**^ | -.600^**^ | -.611^**^ | -.743^**^ | -0.171 |
|  | Sig. (2-tailed) |  | 0.01 | <.001 | <.001 | <.001 | <.001 | <.001 | 0.164 |
|  | N |  | 39 | 68 | 68 | 39 | 68 | 68 | 68 |
| TMT_A | Correlation Coefficient |  | -.599^**^ | -.326^*^ | -.404^**^ | .650^**^ | .635^**^ | .730^**^ | .337^**^ |
|  | Sig. (2-tailed) |  | <.001 | 0.012 | 0.002 | <.001 | <.001 | <.001 | 0.009 |
|  | N |  | 30 | 59 | 59 | 30 | 59 | 59 | 59 |
| TMT_B | Correlation Coefficient |  | -.556^**^ | -.391^**^ | -.410^**^ | .637^**^ | .641^**^ | .754^**^ | .363^**^ |
|  | Sig. (2-tailed) |  | 0.001 | 0.002 | 0.001 | <.001 | <.001 | <.001 | 0.005 |
|  | N |  | 30 | 59 | 59 | 30 | 59 | 59 | 59 |
| MMSE | Correlation Coefficient |  | .467^**^ | 0.203 | .436^**^ | -.614^**^ | -.505^**^ | -.657^**^ | -0.008 |
|  | Sig. (2-tailed) |  | 0.004 | 0.104 | <.001 | <.001 | <.001 | <.001 | 0.952 |
|  | N |  | 36 | 65 | 65 | 36 | 65 | 65 | 65 |
| CAG repeats | Correlation Coefficient |  | 0.013 | -0.121 | 0.099 | 0.196 | 0.299 | 0.233 | -.826^**^ |
|  | Sig. (2-tailed) |  | 0.936 | 0.464 | 0.55 | 0.231 | 0.064 | 0.153 | <.001 |
|  | N |  | 39 | 39 | 39 | 39 | 39 | 39 | 39 |
| Gender | Correlation Coefficient |  | 0.139 | -0.02 | 0.101 | -0.112 | -0.124 | -0.165 | 0.089 |
|  | Sig. (2-tailed) |  | 0.4 | 0.87 | 0.411 | 0.497 | 0.313 | 0.18 | 0.468 |
|  | N |  | 39 | 68 | 68 | 39 | 68 | 68 | 68 |

**. Correlation is significant at the 0.01 level (2-tailed).

*. Correlation is significant at the 0.05 level (2-tailed).

Abbreviations: mRNFL, macular retinal nerve fiber layer; GCIPL, ganglion cell-inner plexiform layer thickness; PIN_HD_, normed version of the Prognostic Index for Huntington's Disease; CAP score, CAG-Age-Product; SDMT, Symbol Digit Modality Task; TMT A, Trail Making Test Part A; TMT B, Trail Making Test Part B; MMSE, Mini Mental State Examination; TMS, Total Motor Score; TFC, Total Functional Capacity.
